# Integration of promoters, inverted repeat sequences and proteomic data into a model for high silencing efficiency of coeliac disease related gliadins in bread wheat

**DOI:** 10.1186/1471-2229-13-136

**Published:** 2013-09-17

**Authors:** Fernando Pistón, Javier Gil-Humanes, Francisco Barro

**Affiliations:** 1 Instituto de Agricultura Sostenible, Consejo Superior de Investigaciones Científicas (IAS-CSIC), Córdoba E-14080, Spain

**Keywords:** Silencing, Endosperm-specific, RNAi, Coeliac, Wheat, Gluten

## Abstract

**Background:**

Wheat gluten has unique nutritional and technological characteristics, but is also a major trigger of allergies and intolerances. One of the most severe diseases caused by gluten is coeliac disease. The peptides produced in the digestive tract by the incomplete digestion of gluten proteins trigger the disease. The majority of the epitopes responsible reside in the gliadin fraction of gluten. The location of the multiple gliadin genes in blocks has to date complicated their elimination by classical breeding techniques or by the use of biotechnological tools.

As an approach to silence multiple gliadin genes we have produced 38 transgenic lines of bread wheat containing combinations of two endosperm-specific promoters and three different inverted repeat sequences to silence three fractions of gliadins by RNA interference.

**Results:**

The effects of the RNA interference constructs on the content of the gluten proteins, total protein and starch, thousand seed weights and SDSS quality tests of flour were analyzed in these transgenic lines in two consecutive years. The characteristics of the inverted repeat sequences were the main factor that determined the efficiency of silencing. The promoter used had less influence on silencing, although a synergy in silencing efficiency was observed when the two promoters were used simultaneously. Genotype and the environment also influenced silencing efficiency.

**Conclusions:**

We conclude that to obtain wheat lines with an optimum reduction of toxic gluten epitopes one needs to take into account the factors of inverted repeat sequences design, promoter choice and also the wheat background used.

## Background

Wheat is one of the most important food grains in the world, being processed into bread and many other products. Wheat products not only make substantial contributions to the dietary intake of energy and protein, but also have impacts on human health, both beneficial (providing dietary fibre, minerals, vitamins, phytochemicals) and negative (allergies and intolerances, which appear to be increasing in importance). Of particular interest is the gluten fraction of wheat grain, as this not only imparts unique technological characteristics, but also plays a major role in intolerance and allergy. The gluten proteins represent 80% of the total grain protein in bread wheat (Shewry and Halford, 2002), and comprise two major groups: the glutenins and the gliadins [1]. The glutenins include the high molecular weight (HMW) and the low molecular weight (LMW) fractions, whereas the gliadins can be divided into three structural types: α-, ω-, and γ-gliadins [2].

In recent years, there have been increases in the incidence of wheat allergies and intolerances. Food allergy to wheat affects 0.2-0.5% of the population [3] and is difficult to manage because very many food products contain wheat. Wheat allergies affects adults and children, can cause anaphylaxis and are triggered by the consumption of gluten. Coeliac disease (CD), which is an intolerance to glutens from wheat, rye and barley and occurs in both children and adults throughout the western world at an average frequency of about 1%, with some groups reporting rates five-fold greater in infants compared with adults [4]. Gluten sensitivity is a new pathology of intolerance to gluten [5], which excludes CD and wheat allergy. No accurate estimates on the prevalence of gluten sensitivity are available, but preliminary data for the USA (6% of the population) suggest that it is more frequent in the general population than CD. CD and wheat allergies require a strict gluten-free diet and sufferers from gluten insensitivity aim to reduce gluten intake as far as possible. Therefore, the development of novel gluten-free wheat varieties is a major objective. CD is the most studied of the gluten-related pathologies. It is an autoimmune disorder with genetic and immunological components as a consequence of the ingestion of gluten proteins from wheat and related cereals. The peptides produced in the digestive tract by the incomplete digestion of gluten cause inflammation of the small intestine and villous atrophy. The autoimmune response is a consequence of the deamidation of glutamine residues present in the peptides, by the tissue transglutaminase 2 (tTG2) in the gut mucosa. The deaminated peptides are able to bind to class II human histocompatibility leukocyte antigen (HLA) molecules DQ2 and DQ8, which stimulate T cells, resulting in an inflammatory response in the small intestine that leads to flattening of the mucosa [6]. Isolation and characterization of intestinal T cells from CD patients have revealed several distinct but similar DQ2 and DQ8 epitopes. Although a number of epitopes are derived from glutenins [7], the majority of the epitopes reside in the gliadin fraction [8,9].

Wheat gliadin genes occur in tightly linked clusters, termed blocks, located at complex loci on group 1 and 6 chromosomes [10]. The estimated copy numbers in hexaploid wheat of genes encoding α-gliadins ranges from 25 to 150 copies [11], from 15 to 18 copies for ω-gliadins, and from 17 to 39 copies for γ-gliadins [12]. This high level of complexity [11,12] and the fact that gliadin genes are inherited in blocks make conventional breeding approaches to obtain wheat varieties with reduced content of T-cell stimulatory epitopes very unlikely.

Post-transcriptional gene silencing by RNA interference (RNAi) is based on sequence-dependent RNA degradation that is triggered by the formation of double-stranded RNA (dsRNA), homologous in sequence to the targeted gene [13]. We used this approach to down-regulate the expression of coeliac disease-related wheat gliadin T-cell epitopes [14,15], showing that RNAi technology can be used to obtain wheat varieties free of toxic epitopes and potentially suitable for CD patients. The use of specific inverted repeat sequences and optimal endosperm-specific promoters, which drive the expression of hairpin constructs, are critical factors in achieving effective down-regulation of CD-related gluten proteins and to minimize off-target effects.

In the present study, 38 transgenic wheat lines with different gliadin fractions down-regulated by RNAi were analyzed over two consecutive years The effect of the promoters and RNAi fragments used for silencing, the genotype, as well as the effect of environment on the content of the gluten protein fractions, total protein and starch, thousand seeds weight and SDSS quality test of flour were analyzed.

## Results

In this work we report the assay of 38 transgenic lines with different gliadin fractions down-regulated by RNAi using two different endosperm specific promoters (D-hordein and gammma-gliadins promoter) and two silencing fragments (to target gamma-gliadins and all gliadins fractions) in a randomized complete block design (RCBD). We used the four combinations of promoter and silencing fragment. The following constructs were used: the vector pghpg8.1 has the D-hordein promoter and the silencing fragment against gamma-gliadins; the vector pGghpg8.1 has the gamma-gliadin promoter and the silencing fragment against gamma-gliadins; pDhp-ω/α vector has the D-hordein promoter and the silencing fragment to target all gliadins fractions; the pGhp-ω/α vector drives the silencing fragment to down-regulate all gliadin fractions with the gamma-gliadin promoter. The assay was repeated during two years to evaluate the environment interaction and gluten protein composition, the thousand seed weights, total protein and starch, and SDSS test were evaluated in transgenic and control lines.

### Analysis of protein fractions from gluten

**Figure 1:**
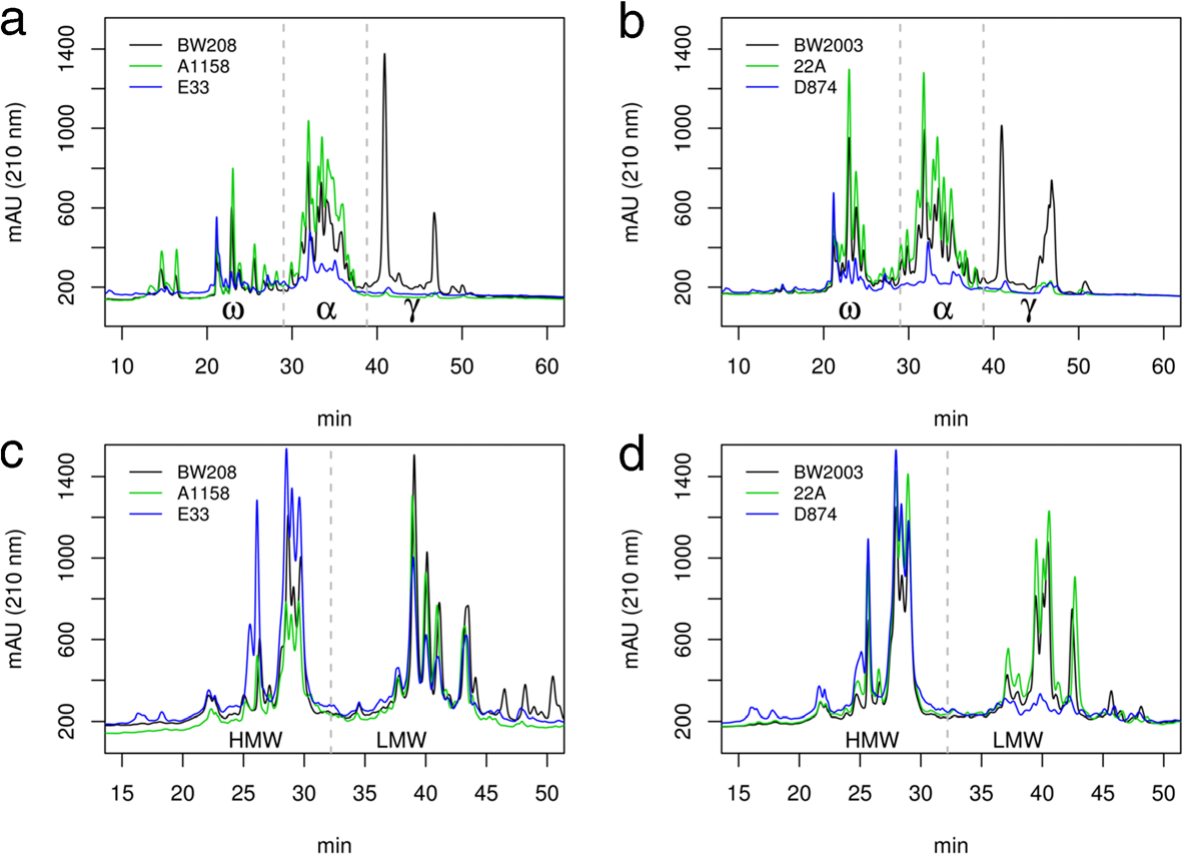
**RP-HPLC chromatograms of gliadin and glutenin extract representing wild-type control and transgenic wheat lines. (a)** Gliadin extracts from BW208 wild-type and BW208 transgenic lines. **(b)** Gliadin extracts from BW2003 wild type and BW2003 transgenic lines. **(c)** Glutenin extracts from BW208 wild type and BW208 transgenic lines. **(d)** Glutenin extracts from BW2003 wild type and BW2003 transgenic lines. ω, ω-gliadins; α, α-gliadins; γ, γ- gliadins; HMW, high molecular weight glutenin subunit; LMW, low molecular weight glutenin subunit. mAU (210 nm), milliunits of absorbance at 210 nm; min, retention time in minutes.

The gliadin and glutenin fractions from the 38 transgenic lines and their respective wild type controls were analyzed by RP-HPLC, where proteins are eluted according to different surface hydrophobicity. The elution order is ω-, α-, and γ-type for the gliadin fraction and HMW and LMW subunits for the glutenin fraction [16,17]. Figure 1 shows the pattern obtained for BW208 and BW2003 controls and four transgenic lines with gliadins down-regulated (Figure 1a and b). The control lines BW208 and BW2003 had different patterns for the gliadin and LMW-GS fractions but the same HMW-GS composition (Figure 1c and d). Chromatograms from transgenic lines were compared with their respective control lines, showing clear differences. Lines A1158 and 22A, transformed with the pghpg8.1 and pGghpg8.1 vectors targeting the γ-gliadins, showed a strong decrease of the γ-gliadin region, whereas the ω- and α-gliadin regions increased the areas of their peaks in the transgenic lines of both genotypes (Figure 1a and b). In the glutenin fractions, these transgenic lines showed an increase of the LMW-GS and HMW-GS (Figure 1c and d). Lines E33, transformed with the pDhp-ω/α and pDhpg8.1 vectors, and D874, transformed with the pDhp-ω/α and pGhp-ω/α vectors, showed a reduction of the α-, ω-, and γ-gliadins region. Both lines E33 and D874 showed an increase in the HMW-GS but line D874 also showed reduction in the LMW-GS fraction (Figure 1d).

We carried out the quantitative analysis of gliadin and glutenin content of all 38 transgenic and wild type control lines by the integration of chromatograms. The efficiency of the constructs on gliadin silencing and their effects on gluten protein was analyzed by a linear mixed model where total protein content (measured by Kjeldahl method) was used as fix covariate, with iTarget and Genotype as fixed-effect factors and Year and Block as random effects (Model 1; Table 1). The *p*-value and explained variance from each effect in the model is reported in Table 1. Total protein, as covariate, had a significant effect on gliadin and glutenin fractions except on the content of LMW-GS and total glutenin content, although its contribution to the overall variance was the lowest of that of the fixed-effects. iTarget was highly significant on the content of gliadin and glutenin fractions, and it was the effect with the higher explained variance, except for the ω-gliadin content. Genotype had a significant effect on the content of ω-, α- and γ-gliadins, and on HMW-GS and LMW-GS, but not on the total content of gliadins, glutenins and Gli/Glu ratio (Table 1). The interaction between iTarget and Genotype (iTarget x Genotype) was also significant for all the gliadin and glutenin fractions, with the exception of ω-gliadins. Together, Genotype and iTarget x Genotype, had lower explained variance than the iTarget effect. The random-effect interaction iTarget x Year was significant for the ω-gliadin content. Year also had a significant effect on the ω-gliadin and LMW-GS contents and on the Gli/Glu ratio, and a borderline significance on total gliadin and HMW-GS contents (Table 1). Except for the contribution of Year to the total variance of the ω-gliadin content (69.77%), the variation of all the variables was mainly due to the fixed effects.

**Table 1:** Significance and percent of explained variance of the fixed and random effects of mixed model for each variable studied

	**Model 1**	**Gliadin**				**Glutenin**									
		**ω**	**α**	**γ**	**Tota**	**LMW**	**HMW**	**Total**	**Gli/Glu**	**Prolamins**	**Non-gluten**	**Protein**	**Starch**	**SDSS**	**Weight 1000**
Fixed	Protein	0.66***	0.69**	1.26**	0.55**	0.06	0.66*	0.05	0.38*	0.65*	2.68***	NA	3.35**	0.16	2.89***
	iTarget	2.59***	32.51***	49.35***	74.58***	34.62***	32.00***	22.56***	16.84***	21.46***	5.34***	6.77***	9.08***	37.54***	33.96***
	Genotype	0.47**	1.02***	5.39***	0.01	5.25**	15.35***	0.05	0.01	0.11	0.80*	0.95·	5.6***	0.05	1.43*
	iTarget x Genotype	0.04	1.17**	1.54*	0.73*	7.11***	1.51*	5.12***	0.84**	1.28*	0.58	2.20	2.17	4.12***	0.59
Random	Itarget x Year	7.89***	1.52	0.38	0.00	0.00	0.05	0.00	5.13	2.79	11.97**	0.00	0.00	0.00	0.00
	Block x Year	3.93***	4.61***	1.81	1.44***	0.19	1.83***	0.62	2.71*	7.03***	12.82***	11.91***	4.46***	0.61	0.00
	Genotype x Year	0.35	0.00	0.00	0.00	1.02	0.05	3.05	2.08	0.00	0.00	0.00	0.00	4.69***	0.00
	Year	69.77**	13.46	0.00	10.86	5.96***	17.31	0.67	30.3*	16.73	4.32	0.00	6.48	3.03	3.20
	Residual	14.31	45.03	40.27	11.82	45.79	31.25	67.89	41.70	49.94	61.49	78.16	68.85	49.80	57.94
	**Model 2**														
Fixed	Protein	0.46	0.54	0.01	0.44	0.47	0.12	0.26	0.49	0.49	73.44***	NA	0.71	0.03	0.41
	iTarget	4.99***	4.63***	6.04***	3.47***	35.99***	30.54***	14.55***	3.30***	3.75***	0.72***	3.66*	5.22**	39.65***	29.29***
	Promoter	0.59	0.32	11.13***	0.68	12.67***	0.34	5.22***	0.35	1.13*	0.22*	2.05	1.26	9.08***	2.38*
Random	Promoter x Year	0.00	0.00	0.25	0.00	0.00	0.00	0.00	0.00	0.00	0.00	0.00	0.00	0.00	0.00
	iTarget x Year	7.67	14.32***	2.69	15.55***	0.00	0.00	0.00	20.17***	11.78**	3.3**	0.00	0.00	0.00	0.00
	Block x Year	17.32***	20.11***	13.14***	19.59***	5.27***	22.03***	20.89***	9.16***	27.37***	7.61***	1.07	6.8**	3.94**	1.98
	Year	0.00	0.00	0.00	0.00	0.17	0.00	0.00	7.67	0.00	0.00	9.13**	0.00	0.00	0.82
	Residual	68.98	60.07	66.75	60.26	45.43	46.96	59.08	58.86	55.49	14.72	84.10	86.02	47.30	65.12

ω, ω-gliadins; α, α-gliadins; γ, γ-gliadins; total, total gliadin content; HMW, high molecular weight; LMW, low molecular weight; Gli/Glu, ratio total gliadin content/total glutenin content; Protein, total protein content in percent of total dry flour weight; Starch, total starch content in percent of total dry flour weight; SDSS, SDS sedimentation test; Weight 1000, 1000 seed weight in grams; iTarget, silencing fragments; C, control line; g, γ-gliadin silencing fragment; o, ω/α-gliadin silencing fragment; go, both silencing fragments; Promoter, the promoter used to drive the silencing fragments; G, γ-gliadin promoter; D, H-hordein promoter; GD, the γ-gliadin and D-hordein promoter. Protein was analized like variable using the Model 1 and 2 removing the covariate. ***, **, *, ·, percent of explained variances are significantly at P < 0.001, P < 0.01, P < 0.05, P < 0.1 respectively.

Levels of factor iTarget were compared by a *post hoc* multiple-comparison analysis (Table 2a). The iTarget ‘g’ (constructs pghpg8.1 and pGghpg8.1) led to a decrease of the γ-gliadin content and to an increase of the HMW-GS and LMW-GS contents relative to control (iTarget C). However, total prolamin content and the Gli/Glu ratio were not significantly affected (Table 2a). The iTarget ‘o’ (constructs pDhp-ω/α and pGhp-ω/α) decreased the content of ω-, α- and γ-gliadins, total gliadins, total prolamins, and Gli/Glu ratio; and increased the content of HMW-GS and LMW-GS. The combination of constructs ‘g’ and ‘o’ (‘iTarget go’) led to a similar effect on the gliadin content than the construct ‘o’ alone, but reduced the levels of all three gliadin fractions and in particular the γ-gliadins fraction. The combination of constructs lead to a lower increment in HMW-GS content than construct ‘o’ alone, but a comparable decrease in LMW-GS content relative to control. Overall, the total glutenin content was compensated (higher HMW-GS and lower LMW-GS) and did not show any difference in comparison to control lines.

**Table 2:** Prolamins and non-gluten contents, gliadins and glutenins contents, total protein and starch contents, SDS sedimentation test and 1000 grain weight of transgenics grouped by silencing fragment and promoter used

	**iTarget**	**Gliadin (μg/mg of flour)**				**Glutenin (μg/mg of flour)**				**(μg/mg of flour)**		**Protein (%)**	**Starch (%)**	**SDSS**	**Weight 1000**
		**ω**	**α**	**γ**	**Total**	**LMW**	**HMW**	**Total**	**Gli/Glu**	**Prolamins**	**Non-gluten**			**(mL · g − 1)**	**(g)**
**a**	C	11.81a	34.61a	23.98c	74.22a	14.62c	9.62d	24.65ab	3.07a	97.45a	32.94a	14.09a	56.17a	12.33c	44.36a
	g	15.46a	41.81a	3.27a	61.21a	17.54a	11.51a	29.44a	2.08a	92.11a	35.56a	15.05a	55.13ab	13.02a	43.98a
	o	5.59b	8.86b	3.79a	18.00b	14.57b	18.31c	32.77ab	0.56b	50.83b	78.63b	14.59a	55.06bc	11.28bc	40.85b
	go	5.82b	7.11b	1.76b	14.20b	10.57b	13.98b	25.22b	0.59b	38.44b	98.36b	15.45b	53.68c	9.86b	38.06b
**b**		**Gliadin**				**Glutenin**			**Gli/Glu**	**Prolamins**	**Non-gluten**	**Protein**	**Starch**	**SDSS**	**Weight 1000**
	**Promoter**	**ω**	**α**	**γ**	**Total**	**LMW**	**HMW**	**Total**							
	D	1	0.74	0.15c	0.58	1.04a	1.62	1.26a	0.46	0.76a	1.00a	1.07	0.98	0.93a	0.90b
	G	0.84	0.65	0.07a	0.49	0.95b	1.63	1.22a	0.4	0.68ab	1.00ab	1.06	0.97	0.93a	0.94a
	GD	0.79	0.61	0.01b	0.44	0.65c	1.52	1.02b	0.43	0.60b	1.01b	1.11	0.96	0.75b	0.92ab

(a) iTarget, silencing fragments; g, γ-gliadin silencing fragment; o, ω/α-gliadin silencing fragment; go, both silencing fragments; ω, ω-gliadins; α, α-gliadins; γ, γ-gliadins; total, total gliadin content; HMW, high molecular weight; LMW, low molecular weight; Gli/Glu, ratio total gliadin content/total glutenin content; Protein, total protein content in percent of total dry flour weight; Starch, total starch content in percent of total dry flour weight; SDSS, SDS sedimentation test; Weight 1000, 1000 kernel weight in grams. (b) Promoter, the promoter used to drive the silencing fragments; C, control line; G, γ-gliadin promoter; D, H-hordein promoter; GD, the γ-gliadin and D-hordein promoter; the values in the promoter table are means of the normalized values relative to control lines (transgenic value/control value). Values within the same column followed by the same letter are not significantly different at P < 0.05 multiple comparison of means test. Variables with values without a letter are not significantly different.

To compare the effect of the two promoters used for the silencing of gliadins, the values from transgenic lines, relative to their wild type control lines, were transformed inside each Block of the experimental design (e.g. value from transgenic line of genotype BW208 of Block 1 from year 2010/control line value of genotype BW208 of Block 1 from year 2010). The fixed-effects were total protein as covariate, iTarget and Promoter; and the random-effects were Block and Year, and their interactions (Model 2; Table 1). The Promoter had a significant effect on the γ-gliadin, LMW-GS, total content of glutenins and prolamins. Furthermore, the Promoter effect explained higher variance (in comparison with the mains factors) in the γ-gliadin content, and explained also high variance in the LMW-GS and total glutenin contents.

Promoter factor was compared by a *post hoc* multiple-comparison of means (Table 2b). The Promoter factor has three levels, the constructs driven by the D-hordein promoter (‘D’), the γ-gliadin promoter (‘G’) and the combination of constructs with ‘G’ and ‘D’ promoters (‘GD’). As shown above, the Promoter factor had effect on the γ-gliadin, LMW-GS, total glutenin and prolamin contents. The strongest decrease in γ-gliadins was seen with the combination of promoters (‘GD’), and the least effect with the D-hordein promoter. The content of LMW-GS was higher with the D-hordein promoter and lower with the combination of both promoters. In addition, when both promoters were combined, they provided lower total glutenin content than using the γ-gliadin and D-hordein promoters independently. The prolamin content was higher with the D-hordein promoter and lower when both promoters were combined (‘GD’).

The Line effect was also evaluated by a mixed model described above (Model 3). The factor Line had a significant effect on the content of all the gliadins and glutenins. To check the differences between transgenic and control lines (each transgenic line against its control genotype), a multiple-comparison of means was made (Table 3). The comparison of means between lines showed an expected behavior according to the iTarget silencing construct used in each case (‘g’, ‘o’ or ‘go’) (see also Table 2b) for the content of ω-, α-, and γ-gliadins, total gliadins, total prolamins and the Gli/Glu ratio. Furthermore, the lines had a higher variability for the content of glutenin (HMW-GS, LMW-GS and total glutenin contents), and these did not follow the same pattern described above for each silencing construct. Thus, we found lines with the silencing constructs ‘g’, ‘o’ and ‘go’ with HMW-GS content equal to their control, in contrast to what was seen when the lines with the same iTarget were analyzed together. Similar observations were made for the content of LMW-GS and total glutenins.

**Table 3:** Prolamins and non-gluten contents, gliadins and glutenins contents, total protein and starch contents, SDS sedimentation test and 1000 seed weight of transgenic and wild-type lines

					**Gliadin (μg/mg of flour)**				**Glutenin (μg/mg of flour)**			**Gli/Glu**	**(μg/mg of flour)**		**Protein (%)**	**Starch (%)**	**SDSS (mL · g − 1)**	**Weight 1000 (g)**
**Genotype**	**Line**	**Constructs**	**Promoter**	**iTarget**	**ω**	**α**	**γ**	**Total**	**LMW**	**HMW**	**Tota**		**Prolamins**	**Non-gluten**				
BW208	Wild type	Control	C	C	11.89	39.59	27.76	78.24	15.51	8.05	23.56	3.31	101.80	26.66	14.21	55.37	12.54	44.25
	A1152	pGhpg8.1 + pDhpg8.1	GD	g	14.98	45.82	1.78***	61.59	18.09*	9.61	27.70	2.24	89.29	45.47	14.90	54.77	13.49	46.79
	A1158	pGhpg8.1 + pDhpg8.1	GD	g	22.56	56.05	2.18***	79.78	17.61	10.01	27.62	2.83	107.40	25.28	14.67	54.83	11.88	46.86
	A1406	pGhpg8.1 + pDhpg8.1	GD	g	18.19	57.84	1.67***	76.70	14.47	11.39***	25.86	2.94	102.56	53.06	17.17***	51.74	9.62***	39.84**
	C655	pDhpg8.1	D	g	16.94	49.20	5.29***	70.44	19.59***	9.38	28.98	2.38	99.41	33.23	14.72	54.00	13.70	41.56
	C657	pDhpg8.1	D	g	25.12	63.24	7.25***	94.61	17.92*	8.88	26.80	3.51	121.41	7.75	14.27	54.48	12.39	44.21
	D445	pDhpg8.1	D	g	41.54*	78.41	2.17***	121.12	21.14***	11.42**	32.55***	3.73	153.68	-7.61	16.16***	52.68	13.55	34.8***
	D577	pGhpg8.1	G	g	18.50	48.90	4.72***	71.13	18.48**	9.79	28.27	2.48	99.39	35.06	14.87	54.46	12.85	42.65
	D623	pDhpg8.1	D	g	16.27	41.91	3.81***	60.99	18.78***	9.81	28.60	2.12*	89.58	45.63	14.92	54.66	12.39	44.50
	D682	pGhpg8.1	G	g	24.02	56.85	4.14***	84.01	19.01***	9.72	28.72	2.95	112.74	15.59	14.20	54.77	13.02	45.36
	28A	pDhp_ω/α	D	o	7.29***	17.13***	5.98***	29.39***	20.22***	16.70***	36.92***	0.82***	66.31**	58.41	13.81	55.21	12.56	39.45***
	28B	pDhp_ω/α	D	o	6.39***	13.46**	5.09***	23.94***	19.95***	18.36***	38.31***	0.60***	62.26***	64.38	13.99	53.92	12.57	42.35
	D770	pGhp_ω/α	G	o	6.25***	10.31***	2.95***	18.50***	17.91*	19.85***	37.76***	0.49***	56.27***	78.30*	14.82	55.03	12.18	39.56**
	D783	pDhp_ω/α	D	o	5.05***	10.87***	4.21***	19.13***	19.6***	17.00***	36.6***	0.53***	55.73***	73.40*	14.26	55.98	12.36	42.56
	D793	pGhp_ω/α	G	o	5.84***	5.78***	1.39***	12.01***	10.01***	14.34***	24.36	0.50***	36.37***	95.60***	14.48	55.68	8.55***	40.66*
	D894	pGhp_ω/α	G	o	5.24***	9.86***	2.87***	16.98***	18.74***	16.37***	35.11***	0.50***	52.10***	78.72*	14.45	54.70	12.08	41.52
	E33	pDhpg8.1 + pDhp_ω/α	D	go	5.88***	8.73***	1.66***	15.27***	15.57	19.86***	35.44***	0.43***	50.71***	90.83**	15.63	54.68	11.94	42.22
	E35	pDhpg8.1 + pDhp_ω/α	D	go	6.47***	10.16***	1.75***	17.38***	16.45	18.45***	34.90***	0.50***	52.28***	80.44*	14.65	54.31	12.27	40.84*
	E39	pDhpg8.1 + pDhp_ω/α	D	go	6.55***	14.88***	1.99***	22.41***	15.47	11.60***	27.07	0.84***	49.49***	93.16**	15.65	52.81	10.25***	37.94***
	E42	pDhpg8.1 + pDhp_ω/α	D	go	5.51***	9.40***	1.29***	15.2***	11.66**	10.07	21.72	0.71***	36.92***	97.86***	14.90	51.78	8.98***	36.51***
	E76	pDhpg8.1 + pDhp_ω/α	D	go	6.38***	3.70***	1.05***	10.12***	6.72***	13.04***	19.76	0.53***	29.88***	103.69***	14.77	53.44	7.18***	33.88***
	E82	pDhpg8.1 + pDhp_ω/α	D	go	6.87***	4.14***	1.04***	11.06***	6.08***	9.29	15.37***	0.76***	26.43***	104.56***	14.46	53.28	6.23***	35.78***
	E83	pDhpg8.1 + pDhp_ω/α	D	go	6.06***	3.15***	1.02***	9.24***	4.99***	8.47	13.46***	0.68***	22.70***	115.74***	15.28	50.91*	6.93***	32.61***
BW2003	Wild type	Control	C	C	12.87	28.15	22.93	62.95	13.93	10.83	24.76	2.53	87.70	39.21	14.01	57.48	11.84	44.65
	22A	pGhpg8.1	G	g	28.25	49.06	3.74***	80.06	18.39***	16.87***	35.25***	2.21	115.31	18.58	14.87	55.85	14.62***	45.03
	22C	pGhpg8.1	G	g	20.79	39.08	2.90***	61.76	17.61***	14.72*	32.33**	1.89	94.10	42.64	15.15	55.68	14.11***	46.71
	24A	pGhpg8.1	G	g	36.51	59.00	5.02***	99.53	18.00**	15.06*	33.06*	3.10	132.59	55.73	16.08·	54.60	13.71**	40.62
	24B	pGhpg8.1	G	g	18.90	34.64	2.75***	55.29	15.47	12.74	28.21	1.97	83.50	53.22	14.68	55.65	11.98	43.99
	C217	pDhpg8.1	D	g	25.01	45.26	9.03***	78.29	15.92	13.64	29.55	2.50	107.85	24.26	14.70	56.56	12.91	43.33
	D598	pDhpg8.1	D	g	16.82	33.07	6.91***	55.80	14.90	11.87	26.76	2.07	82.56	53.87	15.12	56.67	12.21	43.01
	D715	pGhpg8.1	G	g	17.99	34.96	4.94***	56.90	16.99**	14.34·	31.33*	1.81*	88.23	53.77	15.68	56.71	13.75***	47.96
	D716	pGhpg8.1	G	g	18.38	35.48	2.84***	55.70	16.67**	14.62*	31.28*	1.77	86.98	40.84	14.16	54.49	14.04***	48.63*
	D815	pGhpg8.1	G	g	16.95	32.62	2.94***	51.51	15.84	12.61	28.44	1.79	79.95	56.99	14.85	57.19	13.08	38.01***
	D874	pDhp_ω/α + pGhp_ω/α	GD	o	7.17***	5.22***	3.18***	14.58***	6.29***	19.96***	26.25	0.57***	40.83***	95.71*	15.06	54.5	8.95***	39.88**
	D876	pDhp_ω/α + pGhp_ω/α	GD	o	6.17***	5.36***	3.12***	13.65***	5.96***	19.87***	25.83	0.54***	39.48***	95.27*	14.89	54.68	8.94***	39.29***
	E140	pDhp_ω/α	D	o	6.43***	10.49***	6.29***	22.21***	14.42	19.93***	34.35***	0.65***	56.56***	76.2	14.7	56.45	12.24	41.53
	E146	pDhp_ω/α	D	o	8.57***	13.20***	8.15***	28.92***	13.17	21.73***	34.90***	0.80***	63.81**	70.35	14.85	54.61	12.05	42.49
	E122	pDhpg8.1 + pDhp_ω/α	D	go	7.42***	12.57***	3.32***	22.3***	12.62	18.99***	31.61	0.70***	53.91***	103.37*	17.44***	52.19**	11.79	34.32***
	E93	pGhpg8.1 + pGhp_ω/α	G	go	7.03***	7.70***	2.15***	15.88***	8.52***	15.62**	24.15	0.67***	40.03***	98.71**	15.33	54.64	9.38***	38.65***
	E96	pGhpg8.1 + pGhp_ω/α	G	go	7.13***	7.09***	2.03***	15.24***	7.56***	17.38***	24.94	0.62***	40.18***	94.37*	14.89	54.57	9.33***	41.62·

Promoter, the promoter used to drive the silencing fragments; C, control line; G, γ-gliadin promoter; D, H-hordein promoter; GD, the γ-gliadin and D-hordein promoter; iTarget, silencing fragments; g, γ-gliadin silencing fragment; o, ω/α-gliadin silencing fragment; go, both silencing fragments; ω, ω-gliadins; α, α-gliadins; γ, γ-gliadins; total, total gliadin content; HMW, high molecular weight; LMW, low molecular weight; Gli/Glu, ratio total gliadin content/total glutenin content; Protein, total protein content in percent of total dry flour weight; Starch, total starch content in percent of total dry flour weight; SDSS, SDS sedimentation test; Weight 1000, 1000 grain weight in grams. (***, **, *, ·) Means are significantly different to control as determined by multiple comparisons at P < 0.001, P < 0.01, P < 0.05, P < 0.1 respectively.

### Thousand seed weights, total protein and starch, and SDSS assay analysis

Results from SDSS assay, thousand seed weights, total protein and total starch contents, were also analyzed using the mixed model described above (Models 1, 2 and 3; Table 1). The total protein content was analyzed as dependent variable using the same models without the covariate (Table 1). In Model 1 the iTarget had a significant effect in the four dependent variables and it was the effect with the higher explained variance. The covariate effect showed significant differences for total starch and thousand seed weights. Genotype showed significant effect on thousand seed weights and total starch. The interaction iTarget x Genotype led to significant differences in SDSS values. As for the variables analyzed previously (above), the main effect was iTarget, with the higher explained variance for all variables. However, for total starch, the Genotype and total protein together explained almost the same variance as iTarget. The more remarkable random-effects were the Genotype x Year for the SDSS and Year for thousand seed weights. A multiple comparison of adjusted means of the iTarget levels was carried out on these variables (Table 2a). Total protein content of transgenic plants transformed with the iTarget ‘go’ was significantly higher. The transgenic plants with iTarget fragments ‘o’ and ‘go’ had lower total starch contents and thousand seed weights than iTarget ‘g’ and control lines. In the SDSS analyses, the transgenic lines with iTarget ‘g’ showed an increase of SDSS values relative to controls whereas the iTarget ‘go’ showed a decrease and the iTarget ‘o’ a borderline decrease (*p*-value = 0.0557).

The model used to evaluate the effect of the Promoter with the data transformed by the control (Model 2) showed a significant effect of Promoter on SDSS and thousand seed weights but not on Total Starch and Total Protein contents (Table 1). No random factor showed an important effect on the variables. A multiple comparison of adjusted means of the Promoter levels was carried out on these variables (Table 2b). For the SDSS variable, the combination of both promoters was different to the other two, which showed no differences (Promoter ‘D’ and ‘G’). The promoter ‘G’ was different to the promoter ‘D’ and ‘GD’ for thousand seed weight.

### Multivariate analysis of variance (MANOVA) and NMDS

To analyze the data as a whole and to determine which factors have more weight in this dataset, we conducted a nonparametric multivariate analysis of variance (MANOVA). Differences between the two Promoters were tested in the analysis with the data normalized. MANOVA results (Table 4) showed that iTarget, Year, the covariate Total protein, and the interactions Block x Year, and Year x iTarget had a significant effect on the whole data (Table 4; Model M1). The factor iTarget was the main effect and explained 42.4% of the data variability. The interaction Block x Year (5.9%), Year (5.7%), Year x iTarget (3.7%), and the covariate Total protein (1.6%) had minor effect on the data variability.

**Table 4:** Permutational multivariate analysis of variance comparing the whole set of plant traits between total protein, year, genotype, promoter and iTarget of silenced and control plants

**Model M1**	**Df**	**SumsOfSqs**	***P-value***
Protein	1	18320	0.004**
Year	1	65589	0.001***
Genotype	1	3008	0.238
iTarget	3	488888	0.001***
Block x Year	4	68203	0.001***
Year x Genotype	1	392	0.699
Year x iTarget	3	44500	0.002**
Genotype x iTarget	3	10297	0.17
Residuals	223	454532	
Total	240	1153729	
**Model M2**	**Df**	**SumsOfSqs**	***P-value***
Protein	1	11.29	0.005**
Year	1	25.01	0.001***
iTarget	2	265.57	0.001***
Promoter	2	9.17	0.028*
Block x Year	4	81.83	0.001***
Year x iTarget	2	20.91	0.001***
Year x Promoter	2	1.36	0.724
iTarget x Promoter	3	6.17	0.205
Residuals	205	309.67	
Total	222	731	

Df, Degrees of Freedom; SumsOfSqs, sum of squares. *P-value* Significant codes: 0 ‘***’ 0.001 ‘**’ 0.01 ‘*’ 0.05 ‘.’ 0.1 ‘’ 1.

The MANOVA with normalized data showed, in those common factors and covariates, similar results to the previous Promoter model (Model 2). The Promoter factor, introduced in this model, turned out to have a significant effect on the data set (Table 4; Model M2). In terms of explained variance, the effect of Promoter was the least of all significant factors in the model. The two MANOVA models showed similar results to those obtained with the mixed univariate models, except for iTarget x Genotype interaction. This interaction did not show a significant effect in the MANOVA analysis but it was significant in mixed models.

**Figure 2:**
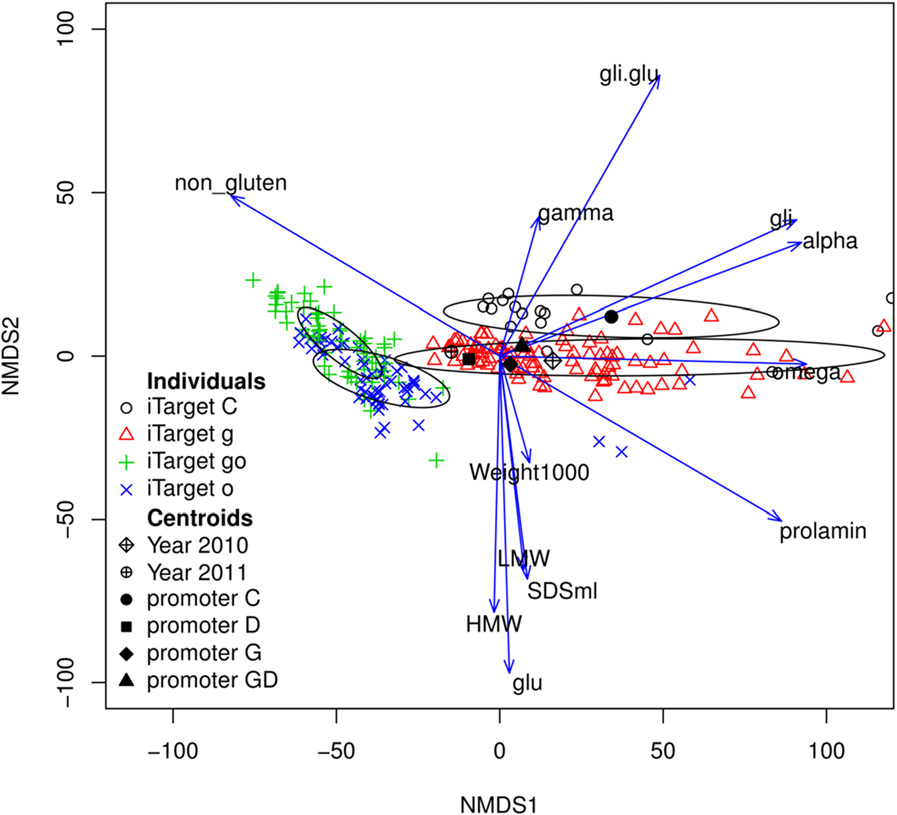
**Non-metric multidimensional scaling (NMDS) graph depicting the relative importance of variables (arrows) explaining the transgenic and wild-type control lines composition.** Ellipses denote the dispersion around the mean values of each iTarget. The individual points are the ordenation of each line and they are colored and symbolized according to their silencing level. The centroids are the average of the levels of the factors indicated. alpha, α-gliadins content; gamma, γ-gliadins content; omega, ω-gliadins content; gli, total gliadin content; HMW, total high molecular weight glutenin subunit content; LMW, total low molecular weight glutenin subunit content; glu, total glutenins content; gli.glu, ratio total gliadins content/total glutenins content; prolamin, prolamins content; non_gluten, non-gluten proteins; SDSml, SDS sedimentation test; Weight1000, thousand seeds weight.

The NMDS ordination plot summarizes the results reported in this paper (Figure 2). The individuals are symbolized and colored according to the inverted repeat sequence used for gliadin silencing (‘g’, fragment targeted to γ-gliadins, ‘o’ chimeric fragment targeted to all three gliadins fraction, ‘go’, combination in different constructs of fragment ‘o’ and ‘g’). Individuals have been surrounded with ellipses to show the dispersion inside each iTarget. Besides, the centroids were plotted for each level of factor Year and Promoter. The most conspicuous feature of the plot was the clustering of individual samples according to the silencing fragment used. The ellipse that grouped together the control samples (iTarget C) was clearly separated from the rest. The same was observed for samples with the fragment targeted to γ-gliadin (iTarget g). Individuals that were transformed with the silencing fragment ‘o’ and the combination ‘go’, overlapped between themselves but none overlapped with control and ‘g’ silenced individuals. The ‘g’ silenced individuals had lower content of γ-gliadins, and a higher content of α-gliadins, ω-gliadins, total gliadins, LMW-GS, HMW-GS, total glutenins, and higher value of SDSS and Weight 1000 (thousand seed weight) than the controls lines. Individuals silenced with the ‘o’ fragment had a lower content of ω-gliadins, α-gliadins, total-gliadins, total prolamins and a lower ratio of Gli/Glu than the control and ‘g’ lines, though, like ‘g’ individuals, they had lower content of γ-gliadins too. Furthermore, these lines (‘o’) showed an increase of the content of LMW-GS, HMW-GS and total glutenin. Finally, individuals with silencing combination ‘go’ were very similar to those with only ‘o’, but the former had a lower overall glutenin content and a lower content of total prolamins. The Year and Promoter centroids did not show much variability, focusing around the center of the plot. However, the year 2010 showed a lower content of ω-gliadins, α-gliadins, total gliadins and total prolamins than year 2011. The Promoter factor levels showed little variation, with the Promoter ‘D’ being the most different from the other two levels (‘G’ y ‘GD’). The control samples and the silenced ‘g’ showed greater variability (greater dispersion of individuals samples) for content of ω-gliadins, α-gliadins and total gliadins.

### Factor interactions analysis

**Figure 3:**
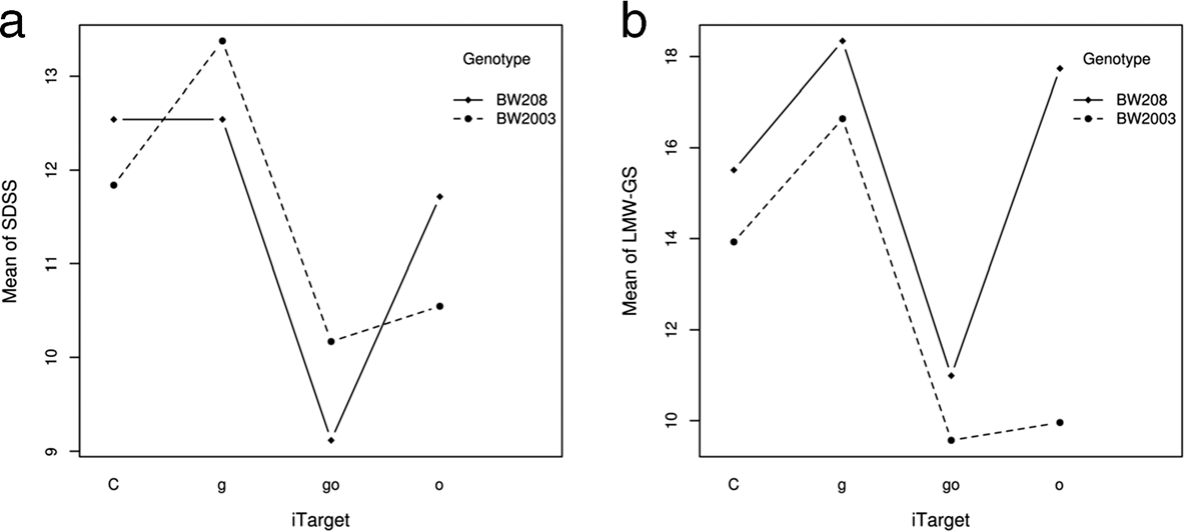
**Interaction plots.** Interaction of the iTarget with the genotype on **(a)** SDS sedimentation test (SDSS) values and **(b)** low molecular weight glutenin subunit content (LMW-GS). C, control lines; g, γ-gliadin silenced lines; o, ω/α-gliadin silenced lines; go, γ- and ω/α-gliadin silenced lines.

The significant interactions in the mixed models reported above are shown graphically (Models 1 and 2) in Figure 3 and Additional file 1 for the variables with significant interaction between iTarget x Year and iTarget x Genotype.

Within iTarget x Genotype interactions, some of the most interesting were those for the SDSS test (Figure 3a) and the LMW-GS content (Figure 3b). In case of the SDSS test, the silenced lines with ‘g’ fragment of the genotype BW2003 had higher SDSS values than control lines but not those of genotype BW208, which showed equal SDSS values than control lines. For both genotypes, transgenic lines with combination of constructs ‘go’ had a marked decrease for the SDSS values in comparison to that of controls ‘C’ and ‘g’ lines. However, this decrease in the SDSS values was not as pronounced for genotype BW208 when the ‘o’ single fragment was used.

iTarget x Genotype interaction on the content of LMW-GS was decreased in lines with silencing ‘o’ of genotype BW2003 but increased in genotype BW208. It is important to highlight that the iTarget x Genotype interaction for the content of LMW-GS had the same pattern as that of SDSS, Total Prolamin and Total Glutenin (Figure 3a, Additional file 1k and i). However, iTarget x Genotype interaction on the content of HMW-GS was increased in both genotypes and iTarget (Additional file 1j).

As shown, for transgenic lines, year 2010 showed higher contents of ω-gliadins, α-gliadins, total gliadins and total prolamins but not of non-gluten proteins. This year effect was more prominent for the lines with silenced ‘g’ (Additional file 1a-f).

## Discussion

In this paper we report 38 transgenic lines from two genotypes with different gliadin fractions silenced by RNAi, using three different promoter combinations and three different silencing construct combinations. The lines were tested during two consecutive years period to evaluate the effect of the promoters, silencing fragments and the environment on the down regulation of wheat gliadins and the effects of that silencing on other storage proteins and quality grain constituents.

Down-regulation of α-, γ-, and ω-gliadins by RNAi is an effective approach to reduce the expression of CD-related gliadin T-cells epitopes [15], which could be the basis for the development of products suitable not only for CD patients but also for other gluten intolerance patients. However, as consequence of this silencing, there is a re-balance of prolamin distribution, leading to the increment of total protein content in some particular lines [15,18], but not as a general effect. In most of the research articles where storage proteins of cereals were silenced by RNAi or mutation, the authors reported either a decrease or no variation in the total protein content [19-24] reported an overall increase of total protein in lines transformed with the ‘g’ antisense fragment, which agree with the data reported in this work. Although the total protein also increases in lines transformed with the silencing fragment ‘go’, this should be interpreted with caution, because the increase in total protein was associated with a decrease in thousand seed weight. It is known that grains that are not completely filled have higher protein content, and a higher embryo to endosperm protein ratio. Thus the increase in total protein in ‘go’ lines may come not only from non-gluten proteins (albumins and globulins) but also from a higher proportion of proteins from the embryo.

Lines transformed with silencing fragment ‘g’ showed a strong reduction of γ-gliadins, which was over-compensated by an increase of α-gliadins, ω-gliadins, HMW-GS and LMW-GS [24]. The term over-compensation is appropriate because the reduction of γ-gliadins results in a higher content of total protein rather than retaining the level of control lines. The reduction of all gliadins, with the silencing fragment ‘o’ or with the combination ‘go’, was accompanied by an increase in HMW-GS and a reduction of LMW’GS, except in line BW2003 with silencing ‘o’ where there was also an increase of LMW-GS. Increased HMW-GS was not enough to offset the lack of gliadins, resulting in the reduction of total content of prolamins, although the total protein remained constant or even increased in the case of the lines silenced with ‘go’ fragments. Therefore, the over-compensation of total protein must be from non-gluten proteins, such as albumins and globulins, as described previously [18] (and as shown by the calculation of non-gluten protein presented in this paper). This compensation effect had been reported in maize and rice, where the silencing of a group of storage proteins leads to a rearrangement of other storage proteins. In maize, the reduction in 22-kDa α-zeins levels by RNAi were compensated by increases of the 19-kDa α-zeins, and vice versa [25]. In the rice mutant line Low Glutelin Content-1 (LGC-1), the content of glutelin was reduced and the contents of other seed storage proteins, including prolamins, were increased [26]. Such up-regulation is not specific to the LGC-1 mutant and it is thought to be a non-specific compensation for the reduction of glutelin. On the other hand, reductions of glutelins and sulfur-rich 10-kDa prolamin levels by RNAi in rice were preferentially compensated by increases of sulfur-poor and other sulfur-rich prolamins, respectively, indicating that sulfur-containing amino acids might be involved in regulating seeds storage protein composition. It could be suggested that transgenic lines, with storage proteins reduced, attempt to compensate total protein content, first with related proteins, and then with unrelated proteins, if necessary. Therefore, the protein compensation could be a selective process because it does not use any kind of protein to compensate. If the compensation is governed by the availability of amino acids, the compensation process may be selectively determined by similarities in the amino acid composition of proteins.

The data presented in this paper allow the detection of differences between the two promoters used for gliadin silencing. Although the Promoter factor explained only part of the variability, it was clear that the γ-gliadin promoter had a higher efficiency which was demonstrated with a better silencing of γ-gliadins, but the efficiency was further increased when both promoters were used in combination. Both the γ-gliadin promoter and, the combination of promoters, led to a decrease of the content of LMW-GS. The contribution to the effectiveness of the promoters that drive a silencing fragment is determined by their expression level during the target expression [27,28]. Results reported by [29,30] concluded that D-hordein and γ-gliadin promoters both had high expression levels in the wheat endosperm but with different expression profiles. The D-hordein promoter was expressed in later stages of grain development than the γ-gliadin promoter. The high efficiency of the γ-gliadin promoter may be due to a higher expression level and/or to a better adjustment with the target genes. However, it is clear that not only the expression level is important, but also a broad expression profile which allows the expression of target genes throughout grain development. This may be what occurred when the combination of the two complementary promoters was used, leading to greater effectiveness.

Among the gliadin fractions, the content of ω-gliadins showed the highest variability being strongly environment-dependent (the factor year explained 69% of the variance). In fact it has been reported that the ω-gliadins modify their expression in response to S deficiency (increasing their expression with low S) [31-35], and N fertilization (the higher input of N the greater accumulation of ω-gliadins) [36-38]. Moreover, the proportions of ω-gliadins increase when grain is exposed to high temperature during grain filling [36,39]. In addition, the high environment-dependence of the ω-gliadin content also could be due to the fact that it is the group which is less efficiently silenced. Therefore, the iTarget factor has less influence on the ω-gliadin content compared with environmental factors.

The iTarget x Genotype interaction for SDSS showed that there was an increase of SDSS value in transgenic lines with iTarget ‘g’ but only for the BW2003 genotype, and a more pronounced decrease of SDSS for genotype BW2003 in comparison with genotype BW208 in iTarget lines ‘o’. Although the iTarget x Genotype interaction for SDSS in iTarget lines ‘o’ has first been analyzed in this work, the different behavior of genotypes with respect to SDSS has already been reported [24]. The latter interaction is associated in turn with the interaction of Genotype x iTarget for LMW-GS. In fact, in the BW2003 lines with a decrease of the SDSS values, this was associated with a decrease of LMW-GS. Moreover, the NMDS ordination graph showed that the LMW-GS content and SDSS value were highly correlated. A similar association between quality parameters and LMW-GS content was also reported by [24], who showed a positive correlation between some mixograph parameters and the SDSS test with an individual LMW-GS peak and total LMW-GS contents.

## Conclusions

iTarget was the main factor that affects the characteristics measured in the the transgenic and control lines analyzed. The silencing is a stable effect over plant generations considered in this study, and produces an efficient and lasting reduction of the different gliadin fractions. Although, the gliadin silencing efficiency is determinated mainly by the factor iTarget, but the promoter, the genotype and the environment factors also affect the gliadin silencing and these factors should be taken into account to achieve a gliadin silencing as high as possible.

The ω-gliadins are the prolamins with the greatest variability, and they are less efficiently silenced. Future work should be directed to the manipulation of this protein fraction to obtain greater and more robust reductions in its content. γ-gliadin silencing is compensated with an increase of α- and ω-gliadins, and to a lesser extent with glutenins. The silencing of all gliadins is compensated by an increase of the glutenin content but also with an increase of non-gluten proteins.

The different promoters tested used alone have a similar efficiency, but the combination of the two promoters with different patterns of expression allows us to cover a wider range of grain developmental stages and thereby higher silencing efficiency. This, may be a general strategy for efficient gene silencing of gene families like gliadins in which constructs under two promoters with different expression profiles, covering as much as possible the expression of target genes during all stages of grain filling.

The results presented will underpin the development of future strategies to achieve more precise and effective silencing of toxic peptides in relation to reducing gluten intolerance pathologies.

## Methods

### Plant material

Twenty-two transgenic lines of *T. aestivum* cv ‘Bobwhite 208’ (BW208) and sixteen transgenic lines of *T. aestivum* cv ‘Bobwhite 2003’ (BW2003) and their corresponding wild-type lines were used in this study. Line BW2003 carries the translocation T1BL.1RS from rye. Four hairpin RNA (hpRNA) vectors were used to down regulate the γ-, α- and ω-gliadins: the pghpg8.1 and pGghpg8.1 vectors down-regulate the γ-gliadins; pDhp- ω/α and pGhp- ω/α vectors down-regulate all gliadin fractions [15]. The constructs pghpg8.1 and pDhp- ω/α contain the D-hordein promoter whereas the constructs pGghpg8.1 and pGhp- ω/α contain the γ-gliadin promoter [29,30]. Transgenic lines A1152, A1158, A1406, C655, C657, D445, D623, C217 and D598 contain the pghpg8.1 vector; lines D577, D682, D715, D716, D815, 22A, 22C, 24A and 24C contain the pGhpg8.1 vector; lines 28A, 28B, D783, E140 and E146 contain the pDhp- ω/α vector; lines D770, D793 and D894 contain the pGhp- ω/α vector; lines D874 and D876 contain pDhp- ω/α and pGhp- ω/α vectors; lines E33, E35, E39, E42, E76, E82, E83 and E122 contain both the pghpg8.1 and pDhp- ω/α vector; lines E93 and E96 contain the pGghpg8.1 and pGhp- ω/α vectors (Table 3). All transgenic lines were previously reported or obtained as described by [15,24] and self-pollinated for four generations to obtain homozygous lines. The seeds used in the assay of second year (year 2011) were from self-pollinated plants of the first assay (year 2010).

### Reversed-phase high-performance liquid chromatography (RP-HPLC)

Gliadins and glutenins were extracted and quantified by RP-HPLC following the protocol reported by [24].

### Thousand seed weight, total protein and starch, sodium dodecyl sulphate sedimentation (SDSS) assay, and non-gluten proteins content calculation

Thousand seed weight (g) was determined for 1000 seeds from each sample. Two measurements were carried out for each sample.

The protein content of whole flour was calculated from the Kjeldahl nitrogen content (%N × 5.7) according to the standard ICC method no. 105/2 [40]. Starch content was determined according to the standard ICC method no. 123/1 [41]. Both parameters were expressed on a dry matter basis.

The SDS sedimentation volume was determined as described by [42]. Three replicates were carried out for each experimental unit.

The non-gluten proteins, expressed in μg/mg, were calculated as follow: [Total protein (%) * ((100 - moisture in%)/100) - (Prolamins content in μg/mg/10)] * 10.

### Experimental design and statistical analysis

All analyses and plots were conducted with the statistical software R version 2.14.1 [43]. The experimental design was a RCBD with three replications of each line and five plants per plot. This experimental design was repeated two consecutive years (2010 and 2011). The randomized block designs were generated with the package agricolae [44]. The variables were submitted to a linear mixed model fitted by restricted maximum likelihood (REML) using function lmer [45]. The models were adjusted using the factors ‘Year’, ‘Block’, ‘Genotype’, ‘iTarget’, ‘ Promoter’ and ‘Line’ and the covariate ‘Total protein’, where ‘Year’ is the year in which the assays were performed (2010 and 2011), ‘Block’ are the three blocks of each RCBD, ‘Genotype’ is the genotype of transgenic and wild types lines (BW208 and BW2003), ‘iTarget’ is the silencing fragment used to silent the gliadins (‘C’ is the control, ‘g’ is the construct to silence γ-gliadins, ‘o’ is the construct to silence all the gliadins, ‘go’ is when the two constructs are used in combination), ‘Promoter’ is the promoter used to drive the silencing fragments (‘D’ is the D-hordein promoter and ‘G’ is the γ-gliadin promoter), and ‘Line’ represent the different transgenic and controls lines used. Factors ‘Year’ and ‘Block’ were considered of random effects, whereas ‘iTarget’, ‘Promoter’ and ‘Line’ were considered of fixed effects. ‘Total protein’ was used as fixed covariate because grain protein content is environment dependent and it has a strong effect on the storage proteins. The lmer model used for testing the effect of ‘Year’, ‘Total protein’, ‘Genotype’ and ‘iTarget’ was: ‘variable ~ Total protein + (1|Year) + (1|Block:Year) + (1|iTarget:Year) + (1|Genotype:Year) + iTarget*Genotype’ (Model 1). Data were normalized against their respective controls before testing the effect of the two promoters (‘D’ and ‘G’) to drive the expression of the silencing fragments. To check the effect of the factor ‘Promoter’, the following lmer model with the data normalized was used: ‘variable ~ Total protein + (1|Year) + (1|Block:Year) + (1|iTarget:Year) + (1|Promoter:Year) + iTarget + Promoter’ (Model 2). The differences between the control and the transgenic lines were assessed using the following mixed effect model: ‘variable ~ Total protein + (1|Year) + (1|Block:Year) + Line’ (Model 3). Residuals were tested for normal distribution and for homogeneity of variances using the model criticism plots generated by the function mcp.fnc (package LMERConvenienceFunctions) [46]. In the cases where the conditions of normality and homogeneity of variances were violated, the Box-Cox transformation was applied (function powerTransform, package car; [47]). p-values for the analysis of variance (or deviance) as well as the amount of deviance explained (%) for each fixed-effect of mixed models were calculated with the function pamer.fnc (package LMERConvenienceFunctions). Post hoc multiple-comparison was carried out with the function glht (package multcomp; [48]).

To identify the relative importance of each factor in the total set of data, we performed a non-parametric multivariate analysis of variance (MANOVA) using the function adonis (package Vegan; [49]). The non-parametric MANOVA model was: ‘variable ~ Total protein + Block%in%Year + Year*Genotype + Year*iTarget + Genotype*iTarget + Line’ (Model M1). The effect of the two promoters in the data set was compared using the following model with normalized data: ‘variable ~ Total protein + Block%in%Year + Year*iTarget + Year*Promoter + Promoter*iTarget + Line’ (Model M2). Nonmetric Multidimensional Scaling (NMDS) ordination plots were generated to demonstrate trends in transgenic and control lines in relation to the variables studied using the function metaMDS (package Vegan). For both analyses (adonis and metaMDS), the method used to calculate the pairwise distances was ‘Euclidean’ with 999 permutations. The package maptools was used to avoid the label overlapping in the ordination plot [50].

## Competing interests

The authors declare that they have no competing interests.

## Authors’ contributions

FB conceived of the study, designed experiments, interpreted the results and wrote the paper. JG conceived of the study, designed experiments, interpreted the results and wrote the paper. FP conceived of the study, designed and carried out experiments, analyzed and interpreted the results, and wrote the paper. All authors read and approved of the final manuscript.

## Supplementary Material

Additional file 1 **Interaction plots of the iTarget with the year (environment) and genotype on the storage proteins contents and ratios.** Only the significant interactions had been plotted. C, control lines; g, γ-gliadin silenced lines; o, ω/α-gliadin silenced lines; go, γ- and ω/α-gliadin silenced lines. Click here for file
