# Supplementary figures and images for: Integration of promoters, inverted repeat sequences and proteomic data into a model for high silencing efficiency of coeliac disease related gliadins in bread wheat
Source: BMC Plant Biol. 2013 Sep 17;13:136. doi: 10.1186/1471-2229-13-136 (PMC3852848; doi:10.1186/1471-2229-13-136)

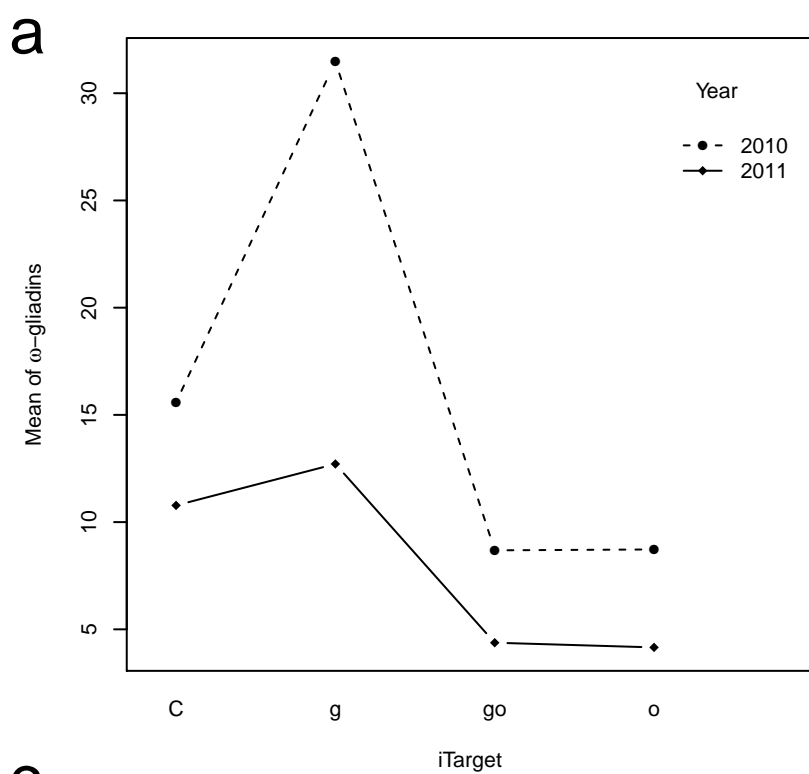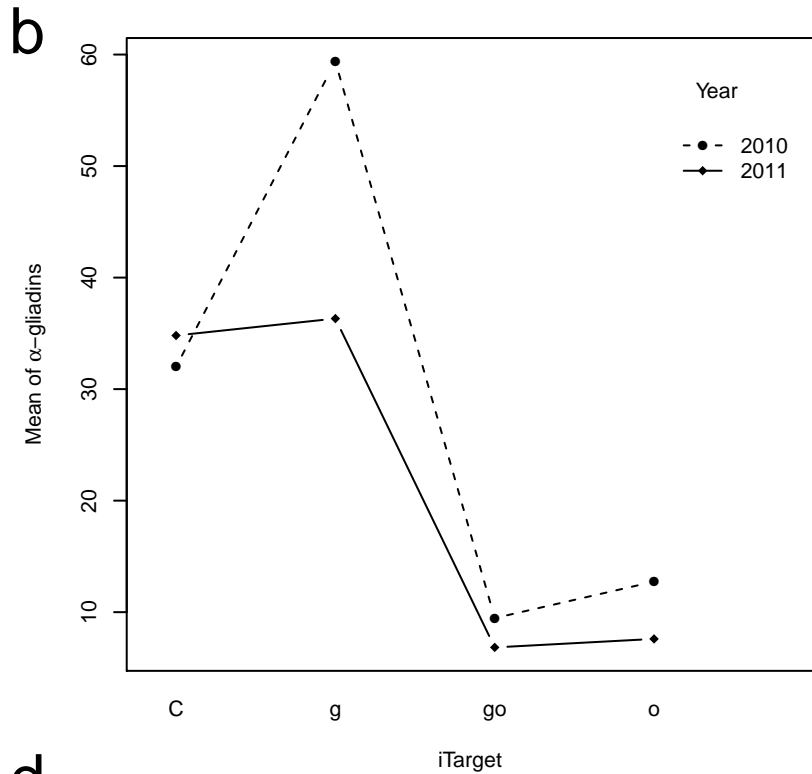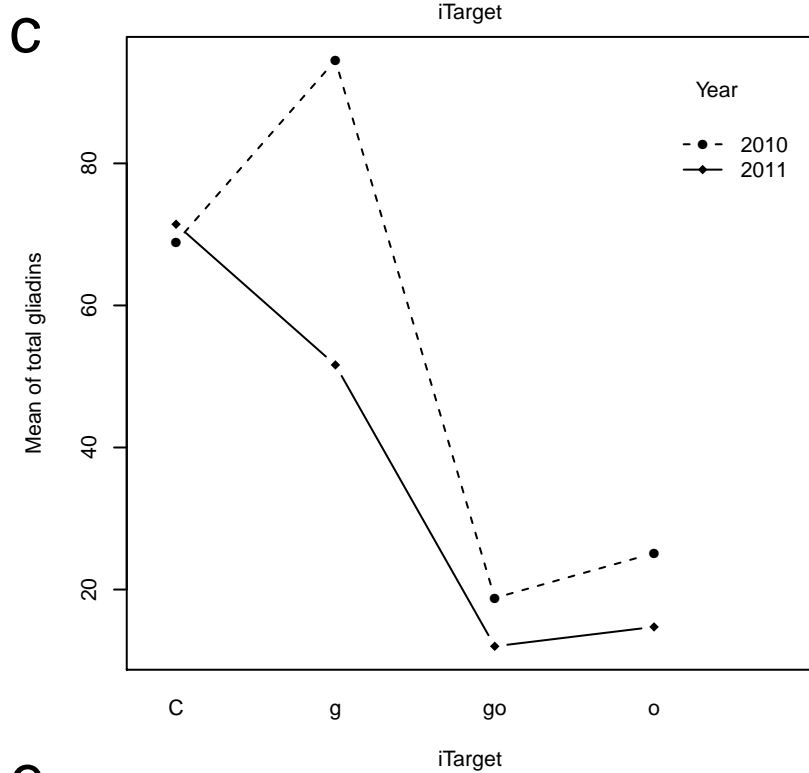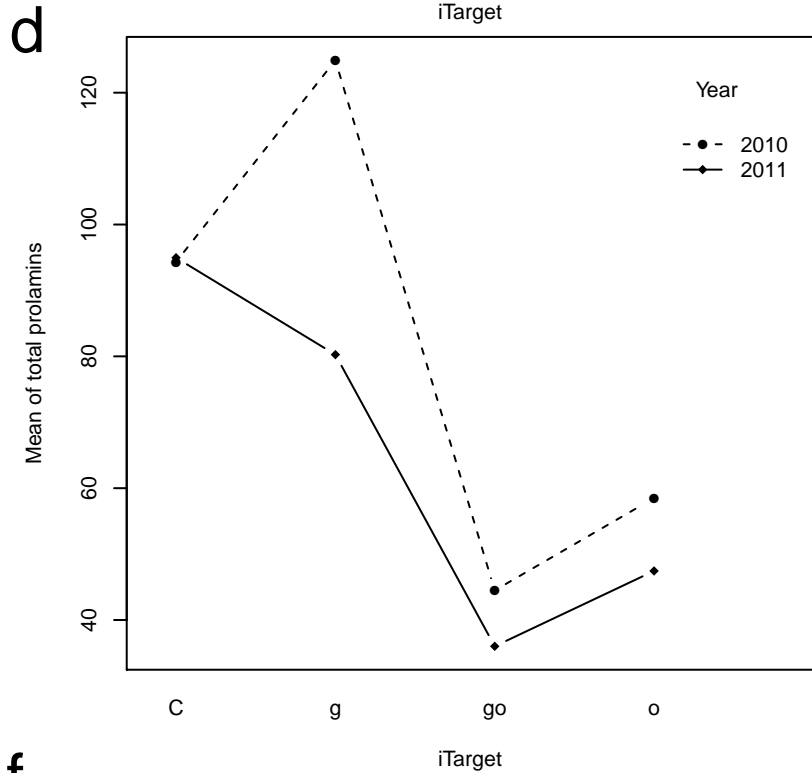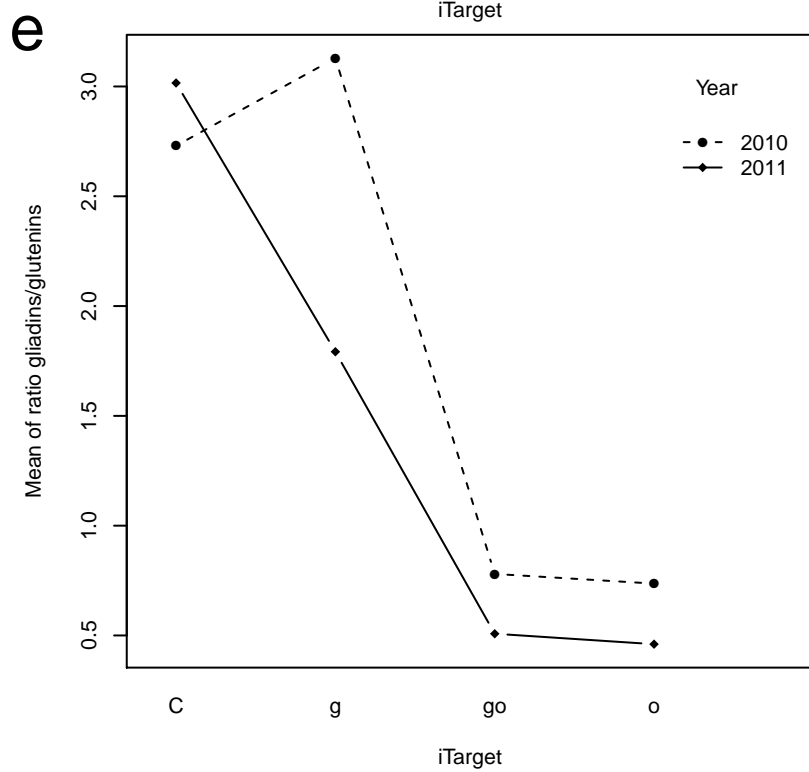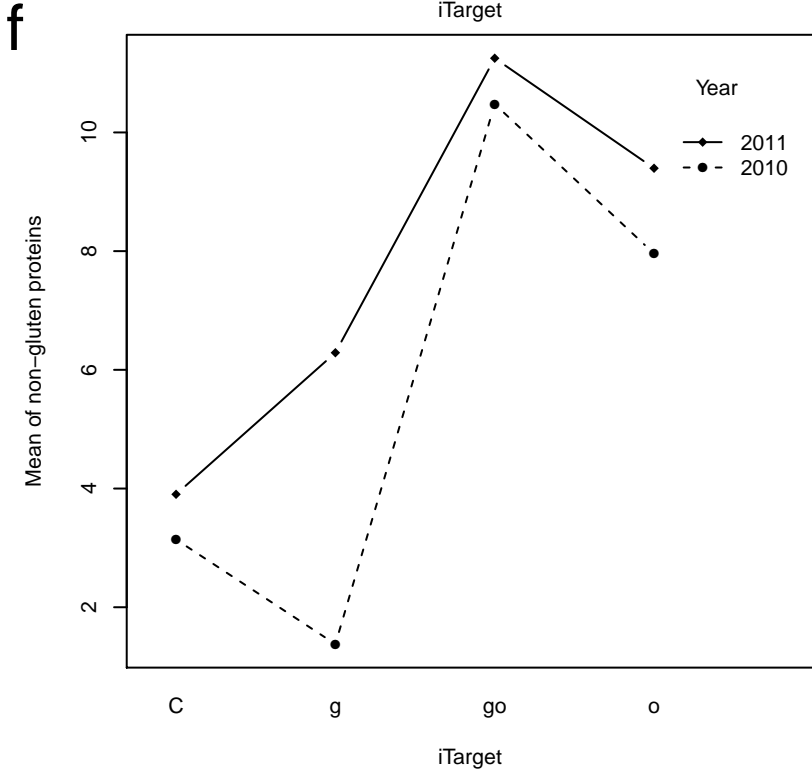

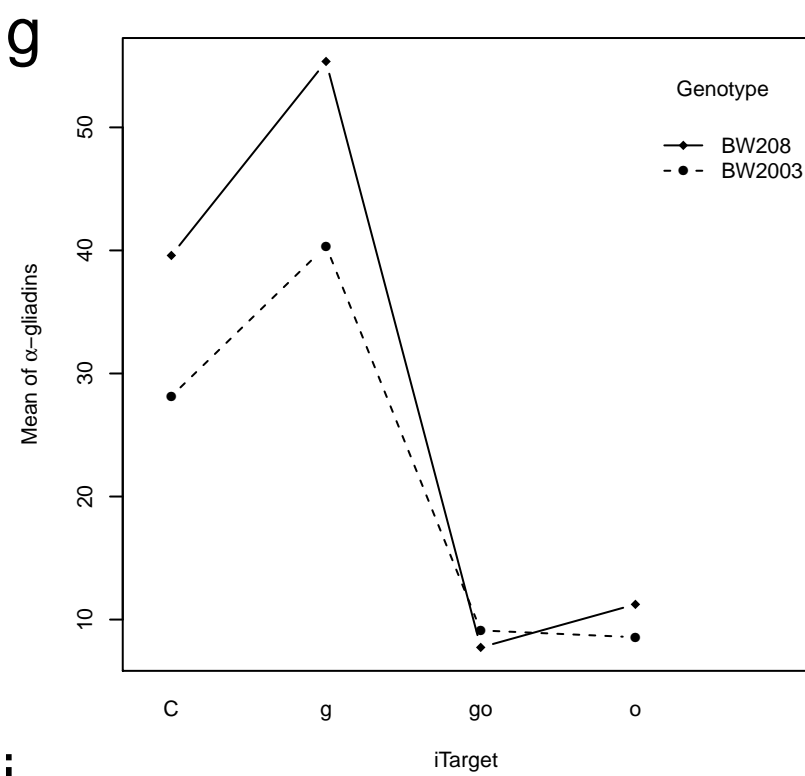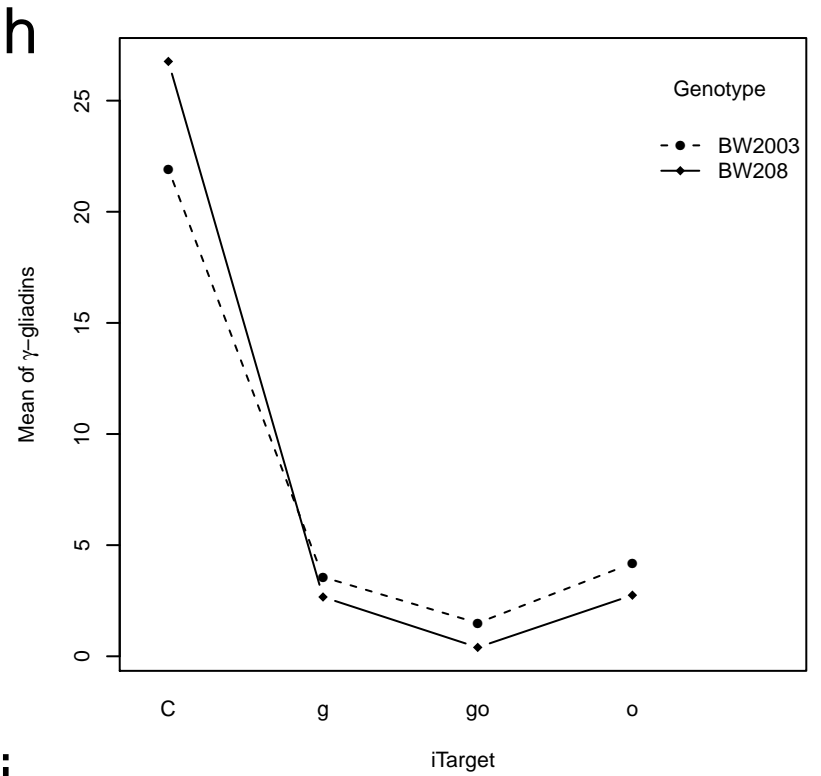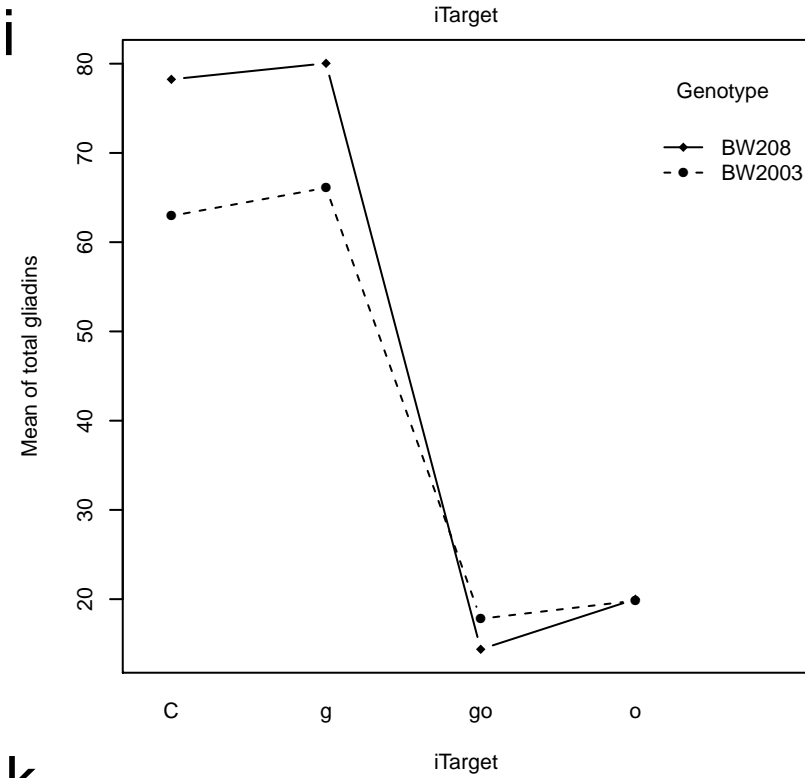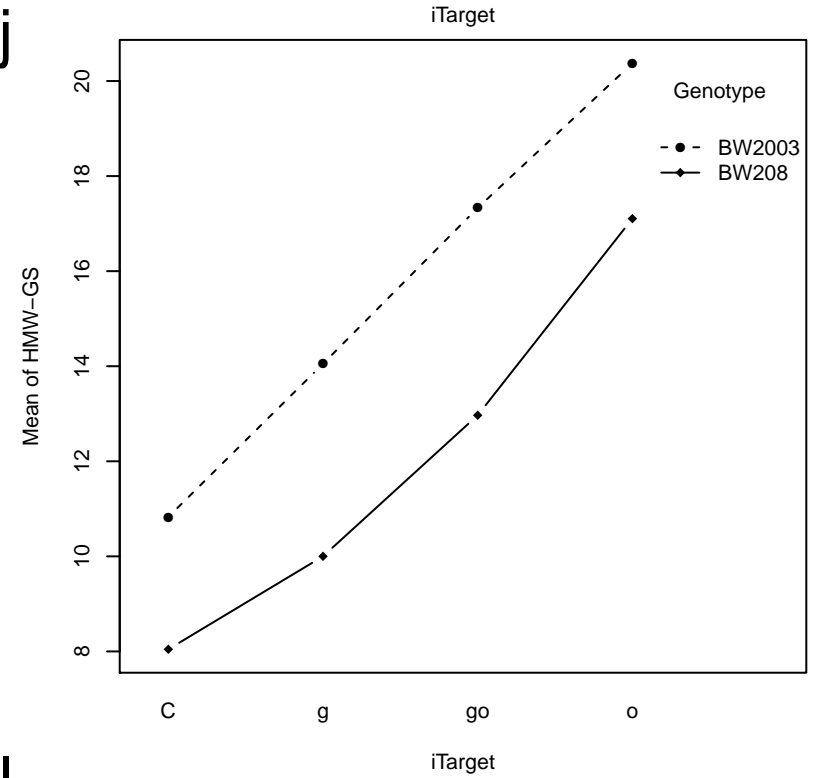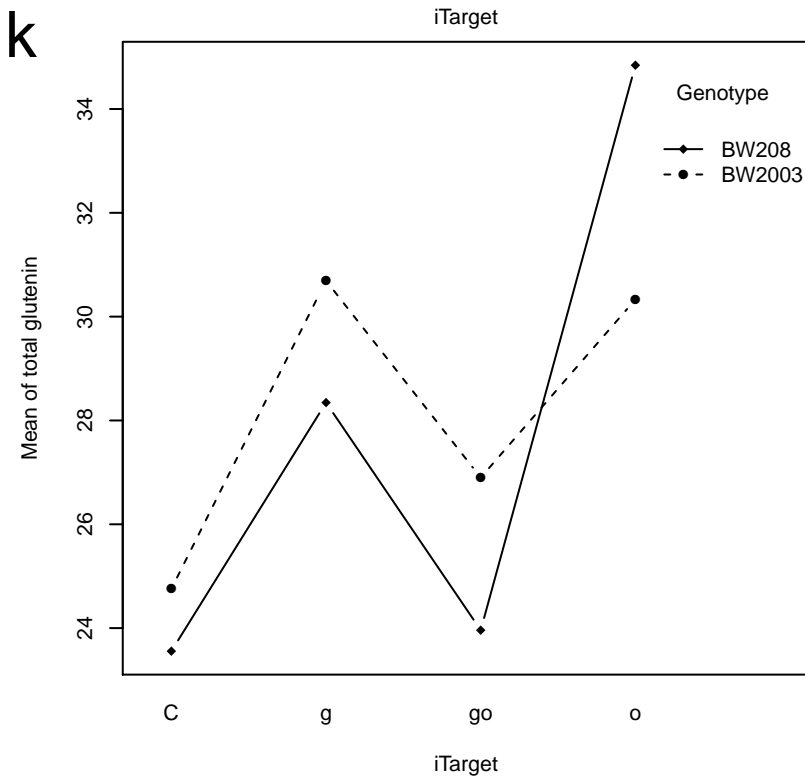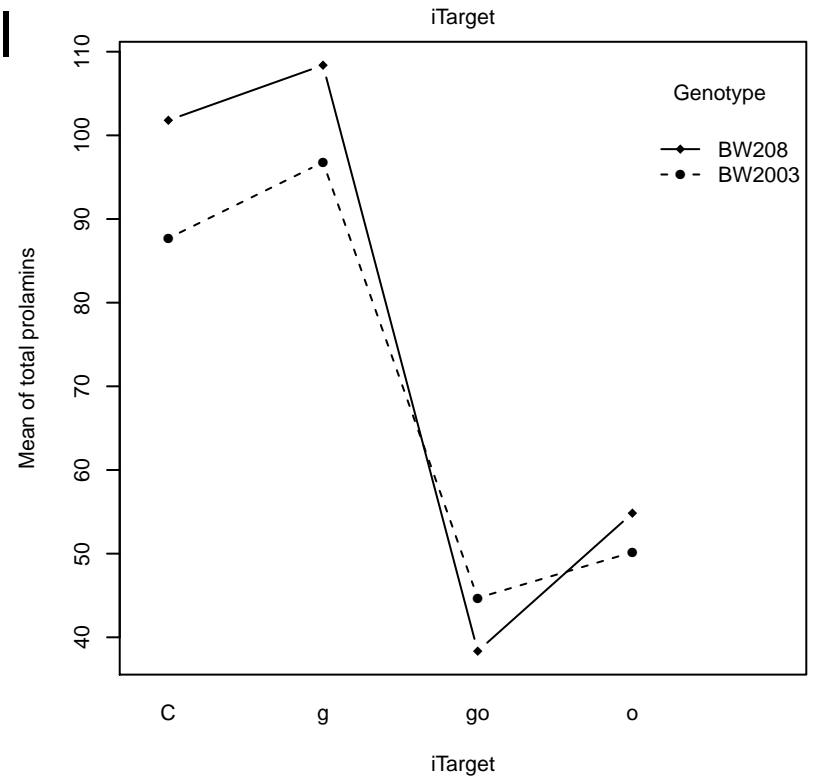

m

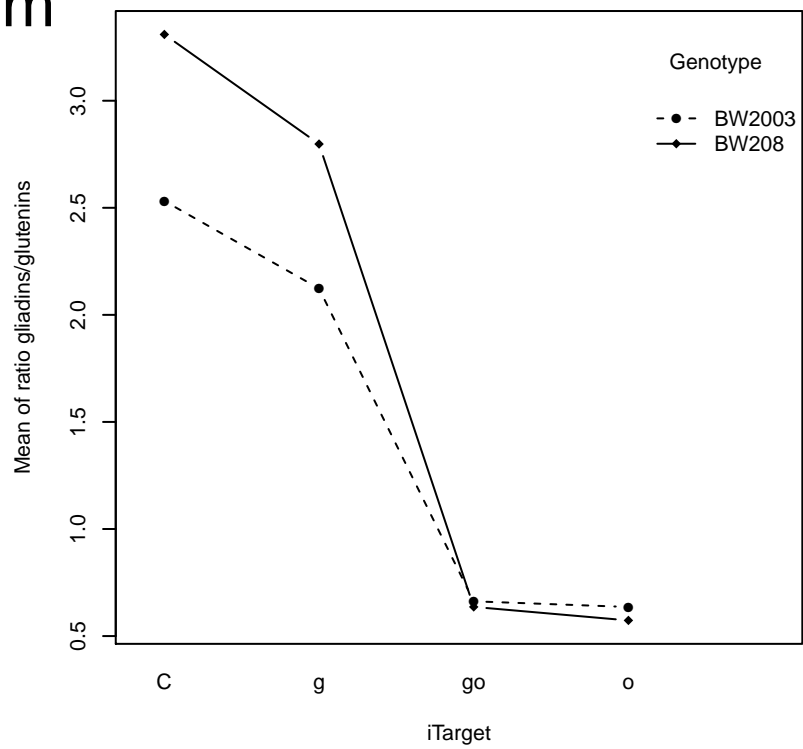

Supplement: Additional file 1 — Interaction plots of the iTarget with the year (environment) and genotype on the storage proteins contents and ratios. Only the significant interactions had been plotted. C, control lines; g, γ-gliadin silenced lines; o, ω/α-gliadin silenced lines; go, γ- and ω/α-gliadin silenced lines. [file 1471-2229-13-136-S1.pdf]
